# From Blinding Vitreous Hemorrhage to 20/20: Multimodal Management of Presumed Tubercular Retinal Vasculitis in a Tuberculosis-Endemic Setting

**DOI:** 10.7759/cureus.90615

**Published:** 2025-08-20

**Authors:** Sindy B Sembiring, Andrea R Silitonga, Rina La D Nora

**Affiliations:** 1 Vitreoretina, SMEC (Sabang Merauke Eye Center) Eye Hospital, Medan, IDN; 2 Ophthalmology and Visual Sciences, Universitas Prima Indonesia, Medan, IDN; 3 Ophthalmology, Cipto Mangunkusumo Hospital, Faculty of Medicine, University of Indonesia, Jakarta, IDN

**Keywords:** anti-tuberculosis therapy, ocular tuberculosis, perivascular infiltrates, posterior sub-tenon steroid, retinal vasculitis

## Abstract

Tubercular retinal vasculitis (TRV) is an ocular manifestation of *Mycobacterium tuberculosis* that can lead to vision-threatening complications such as recurrent vitreous hemorrhage and cystoid macular edema (CME). Even after completing a full course of anti-tubercular therapy (ATT), persistent intraocular inflammation may drive delayed recurrences of CME, highlighting the need for vigilant, escalation-based management. We report a case of a 31-year-old Indonesian woman who presented with sudden-onset hand motion vision in her left eye. Fundus examination was obscured by dense vitreous hemorrhage, and B-scan ultrasonography confirmed an attached retina. Diagnostic workup revealed a positive interferon-γ release assay and apical infiltrates on chest radiography, with no alternative etiology, supporting a diagnosis of presumed TRV. The patient was treated with a standard nine-month course of ATT and tapering oral corticosteroids. Due to progressive retinal ischemia, pan-retinal photocoagulation was performed. Recurrent vitreous hemorrhages and persistent CME necessitated a posterior sub-Tenon triamcinolone injection and 23-gauge pars plana vitrectomy with endolaser at month 9. Fifteen months postoperatively, best-corrected visual acuity had improved to 20/20, with complete CME resolution.

The purpose of this case report is to describe the management of TRV through a progressive escalation of therapy and to outline practical treatment considerations for clinicians working in tuberculosis (TB)-endemic regions. This case also illustrates that early ATT alone may be insufficient to prevent structural complications in TRV. A stepwise, multimodal approach combining systemic therapy, laser photocoagulation, periocular corticosteroids, and timely vitrectomy can restore excellent vision and reduce the risk of recurrent CME. Clinicians managing patients in TB-endemic areas should maintain close optical coherence tomography (OCT)-guided monitoring of CME and be prepared to escalate treatment promptly to prevent irreversible visual loss.

## Introduction

Tuberculosis (TB) is an airborne infectious disease caused by *Mycobacterium tuberculosis* that affects both lungs (pulmonary TB) and other organs (extrapulmonary TB). In 2023, an estimated 10.8 million people were affected globally, marking a 4.6% increase in incidence since 2020. Indonesia ranks second worldwide, with approximately 1.06 million new cases or 394 cases per 100,000 population [1].

Among extrapulmonary manifestations, ocular tuberculosis is a rare but often underdiagnosed form. Ocular involvement occurs in 1.4% of patients with pulmonary tuberculosis, most commonly manifesting as uveitis resulting from the hematogenous dissemination of *Mycobacterium tuberculosis* to the highly vascular uveal tract [2]. Tubercular uveitis (TBU) accounts for approximately 4% of all uveitis cases globally [3], and in Indonesia, it is recognized as a leading cause of infectious uveitis [4].

A severe manifestation of TBU is tubercular retinal vasculitis (TRV), characterized by inflammation of the retinal vessels, primarily veins, with clinical signs such as perivascular (perivenous) inflammatory infiltrates and a risk of vascular occlusion. This clinical picture, in combination with positive immunological testing, such as interferon-gamma release assay (IGRA) or tuberculin skin test (TST), and radiological evidence, in the absence of alternative etiologies, is considered the hallmark of diagnosing TRV in TB-endemic countries [5]. Xie et al. reported a prevalence of 40.5% of retinal vasculitis among cases of presumed ocular tuberculosis [6].

TRV can lead to vision-threatening complications, including neovascularization, which may result in vitreous hemorrhage and tractional retinal detachment, as well as cystoid macular edema (CME). Notably, CME may persist or recur despite adequate antitubercular therapy (ATT), often due to subclinical or residual inflammation [7].

Despite its clinical significance, no standardized treatment algorithm exists for TRV, particularly in guiding the escalation of therapy, including ATT, corticosteroids, laser photocoagulation, or surgical intervention. Most evidence is based on small case series, limiting its generalizability [8]. This case report highlights how a strategically planned, multimodal treatment approach can preserve vision in a patient with TRV complicated by recurrent vitreous hemorrhage and CME, managed in a resource-constrained environment.

## Case presentation

**Figure 1:**
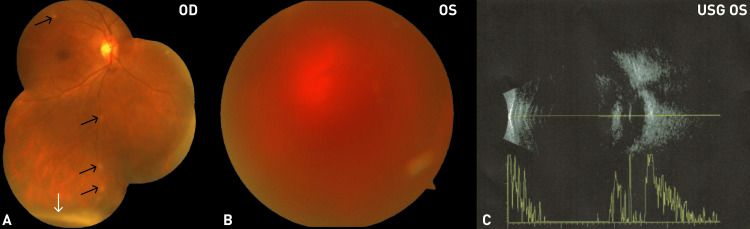
Fundus and B-scan findings at initial presentation (May 2023, Month 0) (A) Fundus photograph of the right eye (OD) showing snowballs (white arrow) and perivascular sheathing and inflammatory infiltrates along the superotemporal and inferior retinal vessels (black arrows); (B) Fundus photograph of the left eye (OS) showing vitreous hemorrhage; (C) B-scan ultrasonography of the left eye revealing dense vitreous opacities with an attached retina.

A 31-year-old Indonesian female presented with sudden visual loss associated with dense floaters, which significantly interfered with her daily activities in the left eye two weeks previously. On presentation (May 2023, Month 0), best-corrected visual acuity (VA) was 20/20 in the right eye (OD) and hand motion (HM) in the left eye (OS), and intraocular pressure (IOP) was normal. There was neither cell nor flare in the anterior chamber nor the vitreous. Dilated fundus examination (DFE) of the right eye and left eye, as well as B-scan ultrasonography, are shown in Figure 1.

A diagnostic workup was performed to evaluate the etiology of the retinal vasculitis. An IGRA was positive, supporting possible *Mycobacterium tuberculosis *infection. Chest radiography revealed a pulmonary infiltrate in the upper right lung, further suggesting systemic TB involvement. In line with the Collaborative Ocular Tuberculosis Study (COTS) consensus guidelines, in TB-endemic regions, the presence of either IGRA or tuberculin skin test (TST) positivity with compatible radiological findings achieves absolute to moderate consensus for initiating anti-tubercular therapy. Therefore, TST was considered optional and was not performed in this case. HIV serology was negative. Serological tests for cytomegalovirus (CMV) and toxoplasmosis were not indicative of active infection. Other tests, including antinuclear antibody (ANA) and syphilis serology, were not performed due to logistical constraints and healthcare system limitations at our center, which made additional testing less feasible once tuberculosis was considered the most likely diagnosis based on available evidence.

Due to the unavailability of intraocular fluid PCR or culture at our institution, microbiologic confirmation could not be obtained. Based on the clinical findings, supportive systemic evidence, and in accordance with the COTS criteria, the diagnosis was classified as presumed tubercular uveitis presenting with retinal vasculitis.

The patient received a nine-month anti-tubercular therapy starting in July 2023, along with three courses of oral corticosteroids at varying doses. Corticosteroids were discontinued thereafter due to steroid-induced arthralgia. A detailed timeline of the clinical course, interventions, and outcomes is summarized in Table 1.

**Table 1:** Timeline of clinical course, interventions, and outcomes over 26 months This table summarizes the monthly progression of best-corrected visual acuity (BCVA), intraocular pressure (IOP), central macular thickness (CMT), episodes of vitreous hemorrhage (VH), anti-tuberculosis therapy (ATT), corticosteroid use, and key ocular procedures in both eyes. OD = right eye; OS = left eye; HM = hand motion; PRP = pan-retinal photocoagulation; PPV = pars plana vitrectomy; RHZE = rifampicin, isoniazid, pyrazinamide, and ethambutol; RH = rifampicin and isoniazid; PSTA = posterior sub-Tenon triamcinolone acetonide

Table 1: Timeline of the Patient’s Clinical Progression																												
Months		0	1	2	3	4	5	6	7	8	9	10	11	12	13	14	15	16	17	18	19	20	21	22	23	24	25	26
Visual Acuity (Snellen Chart)	OD	20/30	20/20	20/30	20/30	20/30	20/40	20/20	20/20	20/20	20/20	20/20	20/20	20/20	20/20	20/20	20/20	20/20	20/20	20/20	20/20	20/20	20/20	20/20	20/20	20/20	20/20	20/20
	OS	HM	HM	HM	20/30	20/50	20/50	HM	20/400	20/100	20/80	20/400	HM	20/100	20/50	20/40	20/20	20/20	20/20	20/20	20/20	20/20	20/20	20/20	20/20	20/20	20/20	20/20
IOP (mmHg)	OD	11	12	16	13	16	12	17	12	14	17	17	18	16	12	17	17	15	15	15	14	13	13	11	13	14	12	13
	OS	8	12	13	13	14	13	14	13	20	25	14	19	19	13	17	21	11	12	13	14	17	14	12	13	12	16	16
CMT (µm)	OD	NA	NA	NA	NA	NA	NA	NA	273	NA	263	NA	266	NA	256	260	254	260	260	267	269	273	290	292	252	281	289	292
	OS	NA	NA	NA	NA	NA	NA	NA	473	NA	554	NA	578	NA	719	473	491	432	547	528	640	471	256	256	468	240	236	237
Vitreous Hemorrhage Occurrence and Clearing in OS		VH			VH Clearing			VH	VH Clearing				VH	VH Clearing and No Recurrence														
ATT		-	-	RHZE	RHZE	RH	RH	RH	RH	RH	RH	RH	-	-	-	-	-	-	-	-	-	-	-	-	-	-	-	-
Oral Methylprednisone (mg)		-	32-16	16	-	48-24	24-16	16-8	8-4	8-4	4	-	32	32-24	24-16	16-8	16-8	16-8	8	4	-	-	-	-	-	-	-	-
Treatment or Surgery											PRP	PRP	Phacomulsification + IOL Implantation + PPV									PSTA Injection (1)			PSTA Injection (2)	

**Figure 2:**
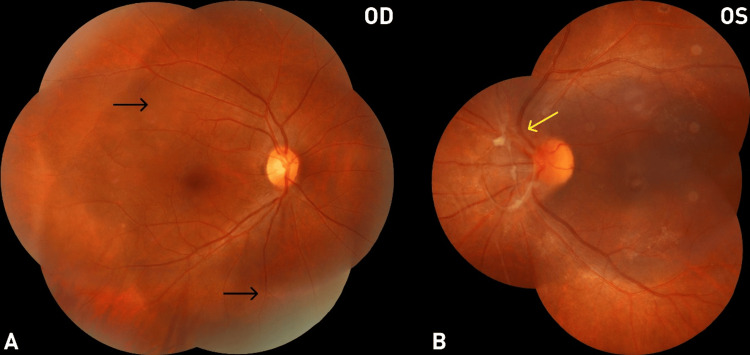
Fundus photograph three months after vitrectomy (July 2024, Month 14) (A) Fundus photograph of the right eye (OD) showing resolution of perivascular sheathing (black arrow), consistent with inactive vasculitis following systemic anti-tubercular therapy and corticosteroids. (B) Fundus photograph of the left eye (OS) showing clearing vitreous hemorrhage with residual fibrovascular membrane at the optic disc (yellow arrow).

Although fundus fluorescein angiography (FFA) was not available at our institution, recurrent vitreous hemorrhage was the strongest clinical indicator of progressive retinal ischemia, suggesting ongoing retinal neovascular activity and prompting the decision for pan-retinal photocoagulation (PRP). PRP was applied to the ischemic peripheral retina of the left eye in two sessions (February and March 2024; Months 9 and 10) using a 577 nm yellow PASCAL laser (Topcon Medical Systems, Inc., Tokyo, Japan) with settings of 300 mW power, 20 ms duration, and 200 µm spot size. In April 2024 (Month 11), the patient experienced a third episode of vitreous hemorrhage, with ultrasound findings suggestive of a tractional process. A 23-gauge pars plana vitrectomy (PPV) combined with phacoemulsification and intraoperative endolaser was performed. No tamponade was used. The vitreous hemorrhage was successfully cleared (Figure 2), with no recurrence observed up to the last follow-up (Month 26).

**Figure 3:**
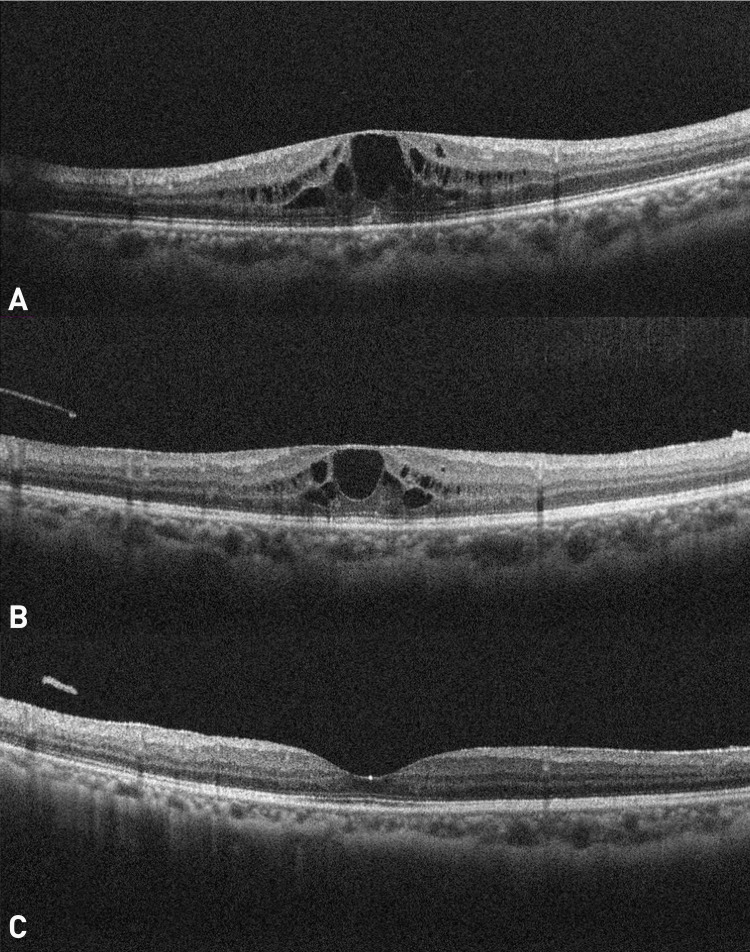
Sequential optical coherence tomography (OCT) images showing cystoid macular edema (CME) and treatment response (A) OCT at February 2024, Month 9, showing cystoid macular edema (CMT: 554 µm) despite ATT and oral corticosteroids; (B) OCT at April 2025, Month 23, showing recurrence three months after the first posterior sub-Tenon triamcinolone acetonide injection (CMT: 468 µm); (C) OCT at July 2025, Month 26 showing resolution after second injection (CMT: 237 µm)

CME was first noted in December 2023 (Month 7), during the final phase of anti-tubercular therapy (ATT) and concurrent oral corticosteroid use. Despite systemic treatment, CME persisted, prompting escalation to periocular steroid therapy. Two posterior sub-Tenon injections of triamcinolone acetonide (40 mg/ml, 0.5 ml) were administered three months apart, in January and April 2025 (Months 20 and 23), following recurrence of CME. At the most recent follow-up in July 2025 (Month 26), best-corrected visual acuity was 20/20 in both eyes, with a quiescent retina and resolution of macular edema (Figure 3). At this visit, she reported full resolution of visual disturbances and subjectively considered her vision completely restored. The patient remains under three-monthly follow-up, including visual acuity testing, intraocular pressure monitoring, optical coherence tomography (OCT) imaging, and dilated fundus examination.

This case highlights the importance of a carefully planned, multimodal escalation strategy to prevent permanent vision loss in patients with tubercular retinal vasculitis, particularly in a TB-endemic region.

## Discussion

TRV, though less common than other forms of TBU, is a vision-threatening condition characterized by retinal inflammation and ischemia. We report a case of presumed TRV in a young, immunocompetent Indonesian woman from a TB-endemic region who presented with severe visual loss due to dense vitreous hemorrhage (VH), later complicated by recurrent CME. Through a stepwise, multimodal approach, including anti-tubercular therapy (ATT), systemic and local corticosteroids, pan-retinal photocoagulation (PRP), and 23-gauge pars plana vitrectomy (PPV), her vision recovered to 20/20.

While most patients respond well to ATT, persistent or recurrent inflammation, including CME and VH, may occur despite adequate systemic treatment. This observation aligns with findings from Gunasekeran et al. and Agrawal et al., who reported treatment failure rates of 14.5% and 14.81%, respectively, in presumed TRV [9,10]. Treatment failure was defined as recurrent inflammation within six months of ATT completion, inability to taper oral corticosteroids to <10 mg/day, or the need for steroid-sparing immunosuppressants [9].

In our case, CME emerged during the ATT course despite concurrent oral corticosteroids and only resolved following posterior sub-Tenon triamcinolone acetonide (PSTA) injections, indicating that systemic therapy alone was insufficient. Recurrence of CME three months after the initial PSTA further supports the hypothesis of chronic subclinical inflammation and blood-retinal barrier dysfunction [7,11]. Escalation to PSTA was undertaken because edema persisted despite a prolonged course of systemic corticosteroids, and the patient developed steroid-related adverse effects. Putera et al. emphasized that a stepwise approach to anti-inflammatory therapy, including escalation to local corticosteroids, resulted in favorable outcomes for both baseline and recurrent uveitic macular edema (UMO), noting that local steroid delivery near the site of inflammation offers enhanced therapeutic efficacy [12]. Although it remains uncertain whether earlier local corticosteroid intervention could have shortened CME duration and lowered the risk of recurrence, localized corticosteroid treatment ultimately achieved durable remission in this case. These findings highlight the importance of long-term OCT monitoring and timely escalation of therapy guided by structural biomarkers. While a dexamethasone implant could have served as an alternative local therapy, its use was limited in our setting by resource constraints and availability [13].

PRP was performed based on clinical signs of extensive retinal ischemia, despite the unavailability of fluorescein angiography. In resource-limited settings, wide-field OCT angiography may serve as a practical alternative. PRP can reduce VEGF production and mitigate the risk of neovascular complications [14]. Gupta et al. reported that timely laser photocoagulation led to regression of neovascularization and helped avoid vitrectomy in most TRV cases [8]. In our patient, recurrent VH was likely multifactorial, involving ongoing neovascular activity, fibrovascular proliferation with vitreoretinal traction, capable of causing vessel rupture even after laser, and suboptimal laser coverage due to limited visualization in the presence of vitreous hemorrhage. Consequently, the patient developed a third episode of VH with B-scan features suggestive of vitreoretinal traction, necessitating surgical intervention.

PPV addressed multiple objectives: clearing dense VH, relieving traction by segmenting the posterior hyaloid, and facilitating endolaser treatment to ischemic areas. It also restored fundus visibility, enabling targeted postoperative monitoring. While observation may suffice in mild or early VH, vitrectomy should be considered in cases with persistent or recurrent hemorrhage beyond six weeks, particularly when tractional elements are suspected [15].

Adjunctive anti-VEGF therapy has also been investigated in TRV. VEGF, a key cytokine among several pro-inflammatory mediators released by activated Müller cells and microglia, disrupts the blood-retinal barrier and contributes to the development of CME [11]. Alcibahy et al. reported favorable outcomes with intravitreal faricimab, a dual Ang-2 and VEGF-A inhibitor, administered following PPV in a case of ocular TB [16]. In our case, anti-VEGF therapy was not administered preoperatively due to B-scan ultrasonography findings suggestive of a tractional component, which raised concern for exacerbating a tractional retinal detachment. Postoperatively, anti-VEGF agents were withheld, as their effectiveness has been questioned in vitrectomized eyes lacking the vitreous body as a drug reservoir [17].

**Figure 4:**
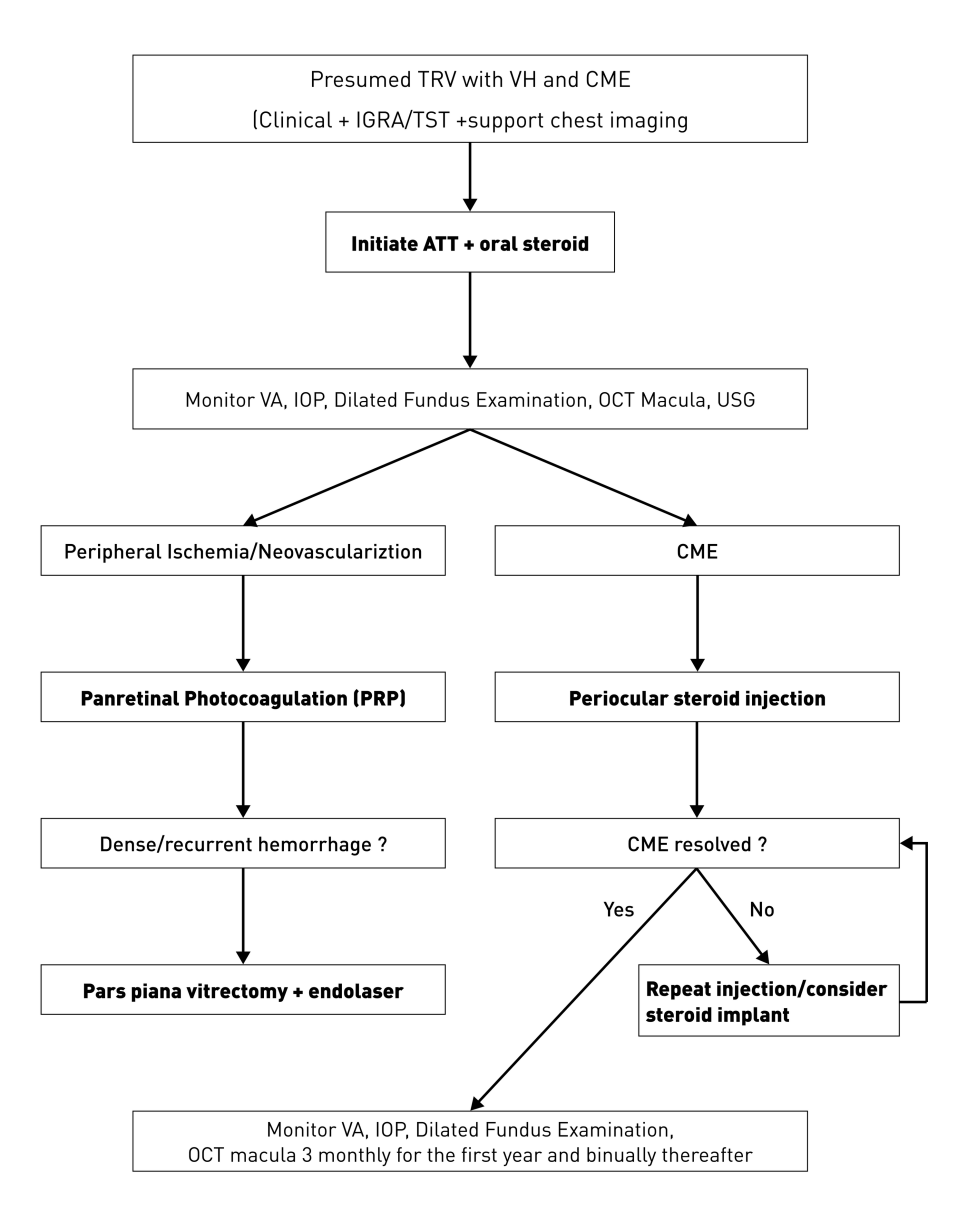
Management algorithm for presumed tubercular retinal vasculitis presenting with vitreous hemorrhage and cystoid macular edema This schematic illustrates the stepwise management approach followed in our case. It is intended as a hypothesis-generating illustration of clinical decision-making in TRV and should not be interpreted as a definitive guideline. TRV: tubercular retinal vasculitis; VH: vitreous hemorrhage; CME: cystoid macular edema; IGRA: interferon-gamma release assay; TST: tuberculin skin test; ATT: anti-tuberculosis therapy; VA: visual acuity; IOP: intraocular pressure; OCT: optical coherence tomography; USG: ultrasonography; PRP: panretinal photocoagulation

This report provides a detailed clinical timeline and serial OCT documentation over 15 months, offering practical insights into escalation strategies in TB-endemic settings (Figure 4). The management sequence illustrated in Figure 4 is drawn from our case and is intended as a hypothesis-generating framework, not a prescriptive guideline. Nonetheless, certain limitations should be acknowledged: the diagnosis was presumptive, as intraocular PCR or culture was not performed; inflammatory biomarkers were not assessed; and the findings are based on a single case, limiting generalizability. Future research should investigate the optimal timing of vitrectomy (early vs. delayed) and the potential role of corticosteroid implants or anti-VEGF agents in recurrent CME associated with TRV.

Lessons learned

This case highlights several important lessons: first, a multimodal treatment strategy combining ATT, corticosteroids, laser photocoagulation, and vitrectomy can achieve sustained visual recovery in TRV, even in TB-endemic, resource-limited settings. Second, OCT proved essential for detecting recurrent CME and guiding timely escalation of therapy. Finally, local corticosteroid delivery offered durable control of inflammation when systemic therapy alone was inadequate.

## Conclusions

In conclusion, prompt initiation of ATT, individualized escalation of corticosteroid therapy, including local administration, and timely surgical intervention can optimize visual outcomes and reduce CME recurrence in TRV. Clinicians practicing in TB-endemic regions should maintain a high index of suspicion for VH recurrence and persistent CME and be prepared to escalate therapy based on structural and inflammatory biomarkers.
